# Quality and usability of home-based record photos to assess vaccine coverage: A case study from the 2022 Democratic Republic of the Congo Vaccine Coverage Survey

**DOI:** 10.1016/j.vaccine.2025.127248

**Published:** 2025-06-20

**Authors:** Dorcas M. Kibeti, Nicole A. Hoff, Sydney Merritt, Aimée M. Lulebo, Jean-Bosco N. Kasonga, Nono M. Mvuama, Christophe L. Luhata, Dalau M. Nkamba, John Samuel Otomba, Amine El Mourid, Aimé M.W. Cikomola, Jean-Crispin Mukendi, Marcellin Mengouo Nimpa, Daniel K. Ishoso, Adèle N. Mudipanu, Deo Manirakiza, Anne W. Rimoin, Didine K. Kaba, Jean K. Nyandwe, M. Carolina Danovaro-Holliday, Paul-Samson D. Lusamba, Eric M. Mafuta

**Affiliations:** a Ecole de Santé Publique, Université de Kinshasa, Kinshasa, Congo; b Jonathan and Karin Fielding School of Public Health, Department of Epidemiology, University of California, Los Angeles, CA, USA; c Programme Elargi de Vaccination, Ministère de la Santé Publique, Kinshasa, Congo; d World Health Organization, Geneva, Switzerland; e Bill and Melinda Gates Foundation, London, United Kingdom; f UNICEF DRC Child survival Office, Kinshasa, Congo

**Keywords:** Vaccination, Home-based records, Democratic Republic of the Congo, Immunization reporting

## Abstract

Home-based records (HBRs) are often considered the “gold-standard” for determining vaccination status - especially for vaccination coverage surveys (VCS). However, data on the quality and usability of HBRs when collected at the source (household) as opposed to photos for recording at a central level is scarce. This study explores the usability of HBR photographs taken during the 2022 VCS of the Democratic Republic of the Congo (DRC). It compares results from analyses of HBR transcribed in the field and those entered from corresponding photos sent to a central server in the assessment of vaccine coverage. This analysis included a random sample of 2768 children aged 12–23 months who had both HBR data entered at the time of the survey and a corresponding photo. Using the card photo, a team of 6 independent researchers transcribed the cards and assessed HBR characteristics based on a predefined set of criteria related to data quality, entry and HBR quality. Data were summarized using proportions; agreement was assessed using concordance and Kappa-Cohen statistics. Over 90 % of selected children had photos considered adequate. Most HBRs met the set quality criteria in terms of physical condition and legibility. The majority (84.6 %) included the Ministry of Health logo and listed all the vaccines in the routine immunization schedule. Concordance and the Kappa-Cohen statistic showed high levels of variability of agreement by vaccine between the two sources of data. This study illustrates that entering vaccine data using photos of HBR at a central level versus at the time of survey is feasible and can provide accurate results with moderate agreement. Further, this approach can also reduce the time per survey in the field, if interviewers are only required to take a photograph. Additionally, having an HBR photo available can be useful for secondary analyses and further training.

## Highlights

• Home-based records (HBRs) allow immunization tracking without recall bias but may slow fieldwork due to added time for data entry.
• Over 90% of 2,768 children randomly selected from the DRC National Vaccine Coverage Survey had quality HBR photos included as part of their interview data.
• Concordance between HBR reported vaccine status when entered in the field and at the central level varied considerably by individual vaccine.

## 1 Introduction

Childhood immunization coverage is an important public health indicator that measures the percentage of children who have been vaccinated against specific diseases, according to a set immunization schedule recommended by health authorities [1,2]. Measuring immunization coverage is necessary for planning national health programs and assessing the performance of health systems at national and subnational levels. However, it is subject to many challenges, primarily record quality and persistent information bias [3,4]. Home-Based Records (HBRs), also known as vaccination or health cards, are widely used in low- and middle-income countries (LMICs) as the only objective reference on immunization history for children [5]. As such, they have been widely used, when available, in studies to evaluate immunization coverage, especially during Vaccine Coverage Surveys (VCS) [6,7].

In most LMICs, HBRs are provided to all children at birth, or when the first vaccine is given, and are kept at home by parents or caregivers. These cards are typically designed at the national level or by partners and are regularly updated as new vaccines are added to the Expanded Program on Immunization (EPI) [8] list of recommended routine immunization (RI). Some HBRs only include information about vaccines, but more and more countries also include information about growth, nutrition and preventative health interventions. Despite the importance of children's HBRs, some studies have shown that they are underused by both caregivers and health professionals, and more importantly, that the information is not consistently or correctly entered when cards are transcribed [[8], [9], [10]]. This negligence can lead to the administration of extra doses, and delays in, or omission of certain vaccines, thereby increasing the risk of vaccine-preventable diseases (VPDs) and complicating efforts in epidemiological surveillance, prevention, and control of infectious diseases among children.

Few studies have examined the quality of HBRs in terms of completeness, legibility, and accuracy of the information contained therein. According to a 2017 South African study on the completeness of HBRs, less than 50 % of HBRs were fully completed within the first six weeks of birth [11]. Similarly, in 2018, a study in Kenya revealed that growth monitoring charts were less frequently available in all examined HBRs [12].

According to the World Health Organization (WHO), in-service training programs for healthcare providers are encouraged to emphasize the importance of completeness and the necessity of simple, neat, and clear writing on HBRs [8]. Additionally, WHO recommends taking pictures of these records during VCS and to evaluate the quality of these photos against criteria used for the design and utilization of HBRs [13]. There is interest in exploring whether there is value if instead of collecting vaccine information directly from HBRs during household visits, data collectors could just take a picture of the HBR and send them to a protected server for transcription at a central level. However, there have been limited studies globally, and none in the Democratic Republic of the Congo (DRC), comparing these two approaches and assessing the usability of a HBR photograph at the central level to collect immunization information.

This study explores the usability of HBR photographs taken during the 2022 DRC VCS and entered at a central level, and compares results from analyses of HBR entered extemporaneously to those entered during household visits as part of the VCS protocol. The DRC VCS follows the WHO recommended methodology for immunization coverage surveys, as adapted by the Kinshasa School of Public Health (KSPH).

## 2 Methods

This descriptive cross-sectional study included the analysis of photos of HBRs from children aged 6–23 months, with a focus on those 12–23 months to assess complete vaccine coverage. These photos were collected during the 2022 DRC VCS as it was conducted in all health zones of the DRC's 26 provinces between February and April 2023. In total, 48,027 HBR photos were collected during the VCS and sent to the central server; of these HBR photos, 41,520 (86.5 %) had valid photos showing the front and back of the card and included the child's identity and vaccine documentation. HBRs included both those from private and public health facilities; the VCS collects immunization data from children in selected clusters regardless of where they were vaccinated. Using a systematic sampling strategy, 6.7 % of the cards (*n* = 2768) from the pool of images considered “valid” (*n* = 41,520) were selected for data extraction; this sampling approach was selected to account for practical considerations by sampling one HBR out of fifteen. HBR data extraction took place in Kinshasa between June and July 2022 and was done by six public health assistants at KSPH who received a three-day training on reading and assessing HBRs.

The study questionnaire was digitized and data was collected using the SurveyCTO application. Data extractors extracted information on the photo quality, card content, model quality, card legibility, the child's socio-demographic characteristics (name, gender, date of birth, weight, and place of birth), the parents' socio-demographic characteristics (name, age, telephone contact), the child's immunization status, and Vitamin A intake. HBR quality was assessed using previously published indicators [14], and HBRs were linked to larger questionnaire responses via a unique identifier barcode included in the picture. HBR content sections were also defined as variables aligned with the sections of the official DRC Ministry of Health issued card, so as to standardize content assessments. Recorded data were downloaded from SurveyCTO server and imported in Stata 17.0 software (StataCorp LLC, Texas, USA) for analysis.

The centrally entered HBRs were paired with 2022 VCS data using a one-to-one option based on unique identifier codes for each child. Data from categorical variables were summarized in proportions for key indicators. Agreement between vaccine coverage data from 2022 VCS data extracted in the field and extemporaneously from HBR photos (one operator) was assessed using Kappa-Cohen test. Vaccine reception was solely compared on a binary (yes/no) for each dose; due to time constraints, no comparison of immunization dates was included. Kappa values range from 0 to 1 with those ≤0.20 indicating slight to poor agreement, 0.21–0.40 fair agreement, 0.41–0.60 moderate agreement, 0.61–0.80 substantial agreement, and those above 0.8 indicating almost perfect agreement [15].

In DRC, the national routine immunization schedule at the time of the VCS was as follows: Bacillus Calmette-Guerin (BCG) vaccine and oral polio vaccine (OPV) at birth; three doses of pentavalent (Diphtheria-Tetanus-Pertussis-*Haemophilus influenzae type b(Hib*)-Hepatitis B), oral polio vaccine, rotavirus and pneumococcal vaccine at 6, 10 and 14-weeks; inactivated polio vaccine at 14 weeks; and measles and yellow fever vaccine at 9 months [16].

### 2.1 Ethical considerations

The current analysis is drawn from the DRC 2022 VCS approved by the Ethics Committee of the Kinshasa School of Public Health (ESP/CE/175/2021). Informed consent was obtained from survey participants. No identifying information from HBRs was recorded.

## 3 Results

### 3.1 HBR Quality

A total of 2768 HBR photos were included in the analysis, of which 2728 (98.8 %) had complete information displayed (identity page and vaccine history page). The majority of HBR images (92.9 %) had good image quality, with no distortion or blurring (72.9 %) or only little blurring that did not impair legibility (20.0 %). The remaining 198 images (7.2 %) could not be read in whole or in part. (Table 1). A foreign object obstructed 25.5 % of the HBR images, but this obstruction only rendered the information needed unidentifiable in 3.5 % of the photos. Furthermore, more than 80 % of the HBR photos showed no signs of physical damage that could reduce the ability to read the information on the card. Furthermore, when evaluating the handwriting and legibility of the completed information, three quarters (75.4 %) were deemed of excellent or good quality handwriting, and 58.3 % of the information included was deemed suitable for the space provided. With regard to the corrections indicated on the cards, approximately 30.0 % indicated errors, but only 4.9 % were correctly marked with the responsible healthcare workers' initials.

**Table 1:** Quality of the HBR and written documentation photos.

Variables	n (%)
**Card image shows complete information (vaccine page and identity page)**	2728 (98.8)
**Image blurred, overexposed or distorted to such an extent that you can't read the text**	
Not blurred or distorted	2014 (72.9)
Slightly blurred or distorted, but still legible	552 (20.0)
Moderately blurred or distorted, some parts illegible	176 (6.4)
Severely blurred or distorted, illegible	22 (0.8)
**Loss of information on the HBR photos due to foreign objects**	
No foreign object obscuring image	2058 (74.5)
A foreign object hides the image, but no information is lost	608 (22.0)
A foreign object hides the image, little information is lost	89 (3.2)
A foreign object hides the image, much information is lost	8 (0.3)
**Presence of evidence of physical damage (discoloration, tearing, creasing, mildew, cutting by rodents, traces of fire, humidity, liquid damage…) on the card**	
No damage	228 (82.5)
Slight damage, but no loss of information	340 (12.3)
Moderate damage, small loss of information	115 (4.2)
Severe damage, significant loss of information	29 (1.0)
**Presence of highlight marks, punch holes or staples, reducing the ability to read information on the card**	
No, no obstruction	2438 (88.2)
Yes, slight obstruction of information	233 (8.4)
Yes, moderate information obstruction	71 (2.6)
Yes, severe information obstruction	21 (0.8)
**Handwriting on the card clear and easy to read**	
Excellent	399 (14.5)
Good	1681 (60.9)
Fair	610 (22.1)
Poor	70 (2.5)
**Encroachment**	
Yes, all answers are suitable for spaces	1611 (58.3)
No, some answers are not suitable for spacing but are still legible	1098 (39.8)
No, some answers do not fit in the spaces, some parts are not very legible	44 (1.6)
Don't know	8 (0.3)
**The card contains errors and corrections clearly marked**	
Errors marked and new values legible and initialed	134 (4.9)
Errors marked and new values legible, not initialed	645 (23.4)
Corrections unclear/Not legible	54 (2.0)
Not applicable, no obvious corrections	1927 (69.8)
**Card shows pre-printed fields for recording required information**	2693 (97.6)

### 3.2 HBR Models and content

**Fig. 1:**
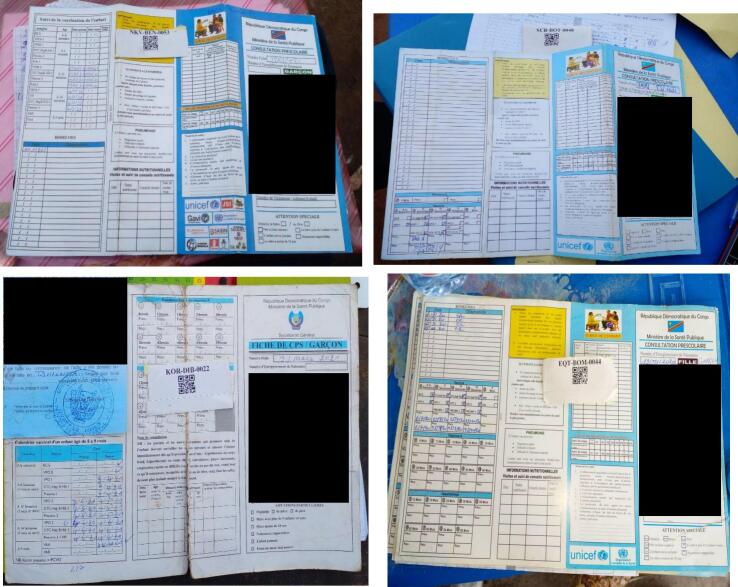
HBR models seen during 2022 DRC VCS. A) Older version of HBR that does not list all current vaccines, B) Older version of HBR that does not have space for dates of each individual vaccine, C) Correct version of HBR with all vaccines and space for dates of each vaccine, D) Older version of HBR missing some current vaccines and no space for individual vaccine dates.

Of the HBR photos in this analysis, 100 % were written in the DRC's official language, French, and 74.0 % of them were printed with the Ministry of Health logo, with the immunization schedule placed at the top, and separate spaces for the dates of each vaccine (Table 2). The remaining HBR records had some variation of these elements, with some cards having outdated vaccines, no space for individual dates, or no Ministry of Health logo. Examples of selected HBR variations are presented in Fig. 1, Fig. 1c presents the most updated HBR card with the correct vaccines listed for 2021 (the year most that children enrolled in the 2022 VCS were vaccinated).

**Table 2:** Models of the home-based vaccine card photos.

HBR Model	n (%)
Printed card with the Ministry of Health logo, the vaccination calendar is positioned at the top, with separate date ranges for each vaccine.	2044 (74.0)
Printed card with the Ministry of Health logo, the vaccination calendar is positioned at the top, with a single date range for all vaccines supposed to be given at the same time.	3 (0.1)
Card printed with the Ministry of Health logo, the vaccination schedule is placed in the middle, with a single date range for all vaccines supposed to be given at the same time.	183 (6.6)
Printed card with the Ministry of Health logo, the vaccination schedule is placed at the bottom, with a single date range for all vaccines recommended at the same time.	111 (4.0)
Photocopy of card with the Ministry of Health logo, vaccine schedule at top, with separate date ranges for each vaccine	353 (12.8)
Photocopy of card with the Ministry of Health logo, the vaccination calendar is placed at the top, with a single date range for all vaccines recommended at the same time	3 (0.1)
Photocopy of the card with the Ministry of Health logo, the vaccination schedule is placed in the middle, with a unique date range for all vaccines recommended at the same time.	37 (1.3)
Photocopy of the card with the Ministry of Health logo, the vaccination schedule is placed at the bottom, with a single date range for all vaccines recommended at the same time.	10 (0.4)
Printed card without Ministry logo	5 (0.2)
Other card or photocopy of card	14 (0.5)
**HBR Content**	**n (%)**
Card contains information on vaccines recommended by the national immunization schedule (name of each vaccine)	2727 (98.8)
The card contains information on each disease against which the child is protected by each vaccine in the vaccination schedule	929 (33.7)
The card contains structured spaces (ranges) to record the dates of receipt of each vaccine and each dose	2186 (79.3)
Card contains information on the health facility where vaccines are administered	2527 (91.5)
Card contains space to record date of next routine immunization visit	2605 (94.2)
Card contains enough space for a few notes or remarks from the healthcare provider	2523 (91.3)
Card contains space for known allergies and adverse reactions	714 (25.9)
Vaccination card is written in the country's official language (French)	2759 (99.8)
The card has a flexible design to allow for future changes in the national immunization schedule	2073 (75.0)
Card has structured field format for data recording including checkboxes and appropriate date format	2587 (93.7)
Card provides separate information on recommended ages for each vaccine in the immunization schedule	2456 (88.9)
Card clearly and prominently displays date of next routine immunization visit	2321 (83.9)

Almost all HBR photos contained information about vaccines recommended by the national immunization schedule (98.8 %). While not all cards had the most updated vaccine schedule, 75.0 % of the HBRs had a flexible design to accommodate future changes in this schedule. Approximately seven of ten HBR photos indicated that cards contained structured spaces (sections) to note the dates of receiving each vaccine and dose. More than 90 % of the HBR photos showed that cards had enough space available to note the date of the next routine immunization visit, as well as sufficient space for some notes or remarks from the healthcare provider. However, most cards did not have enough space to document known allergies and adverse reactions (74.1 %). Over 90.0 % of the HBRs had the space to note where vaccines were received and the majority of cards prominently displayed the date of the next routine immunization visit.

### 3.3 HBR Completion of Child and Parent's socio-demographic characteristics

Almost all HBR photos indicated the full name of the child (90.5 %), sex of the child (98.7 %), date of birth of the child (97.9 %) and child place of birth (97.5 %) but included the record number and registration number in only 47.3 % and 32.1 % of the HBR photos, respectively. The record number indicates an internal record for each health facility while a child's registration number is an official birth indicator as collected by the national government. Of note, about one in ten children were born at home (Table 3).

**Table 3:** Child and parent's socio-demographic characteristics recorded in HBR photos.

Child Variables	n (%)
The child's record number is filled on the card	1309 (47.3)
The child's registration number is filled on the card	884 (32.1)
The child's full name is indicated on the card	2506 (90.5)
The child's sex is indicated on the card	2732 (98.7)
The child's date of birth is marked	2711 (97.9)
The child's birth weight is marked	2103 (76.0)
The child's place of birth indicated	2685 (97.5)
The child is born at home	182 (7.2)
The child's province is indicated	2630 (95.3)
The health zone (health district) in which the child is located is indicated	2708 (98.0)
The health area (sub-district) in which the child lives is indicated	2675 (96.6)
The child's position in relation to the health zone is indicated (living in or out of current health zone).	1028 (37.1)
The child's position in relation to the health area is indicated (living in or out of current health area)	1000 (36.1)
Residence, if different than the Health Area where the card was issued	81 (8.1)
**Parental/Guardian Variables**	**n (%)**
Mother's name is marked	2679 (96.8)
Mother's age is marked	2215 (80.2)
Father's name is marked	2680 (96.8)
Child's home address is marked	2529 (91.5)
A space is provided for parents' telephone number	2264 (82.0)
Special attention space is provided to the child to note important information	2479 (89.6)

Regarding parent or guardian contact information, almost all HBRs in the analysis had a section designated for the parents' address and telephone numbers and a section for special attention regarding the child. Over 95.0 % of the HBRs indicated the parents' names and home address (Table 3).

### 3.4 Agreement between vaccine coverage data from HBR photos and data from VCS entered at the household

Vaccine data recorded from the HBR photos at the central level were compared to data recorded from the actual HBR entered during household surveys by local interviewers. Agreement ranged between 49.8 % for the third dose of rotavirus vaccine up to 77.5 % for the first dose of Pentavalent vaccine (Table 4a, Table 4b). With each additional dose of multiple-dose schedule vaccines, concordance between sources decreased. Kappa-Cohen statistics suggested slight to poor agreement for all doses (<0.20).

**Table 4a:** Two by two tables showing agreement of vaccination status between data from home-based vaccine cards photos and data form vaccination home-based record per vaccine dose (n = 2768), DRC VCS 2022 for some vaccines.

	Having received the vaccine according to HBR		
Having received the vaccine according to the VCS Data	No	Yes	Total
*BCG*			
No	379	597	976
Yes	619	1173	1792
Total	998	1770	2768
*Penta 1*			
No	62	315	377
Yes	142	1514	1656
	204	1829	2033
*Rota3*			
No	556	809	1365
Yes	210	458	668
Total	766	1267	2033

**Table 4b:** Agreement of vaccination status between data from home-based vaccine cards photos and data form vaccination home-based record per vaccine dose (n = 2768), DRC VCS 2022.

Vaccine	Concordance (%)	Kappa-Cohen (for agreement between data HBR photos and data VCS)	*p*-value
OPV 0	58.9 %	0.0818	<0.05
BCG	56.1 %	0.0427	<0.05
OPV 1	76.1 %	0.0729	<0.05
OPV 2	65.3 %	0.0705	<0.05
OPV 3	58.8 %	0.0964	<0.05
IPV	57.8 %	0.1102	<0.05
Penta 1	77.5 %	0.0957	<0.05
Penta 2	65.8 %	0.0777	<0.05
Penta 3	58.6 %	0.0919	<0.05
PCV13 1	76.6 %	0.0900	<0.05
PCV13 2	64.8 %	0.0729	<0.05
PCV13 3	58.6 %	0.0976	<0.05
ROTA 1	67.5 %	0.0487	<0.05
ROTA 2	59.0 %	0.0598	<0.05
ROTA 3	49.9 %	0.0756	<0.05
MCV 1	55.7 %	0.0772	<0.05
YF	55.4 %	0.0715	<0.05

OPV: Oral polio vaccine.

IPV: Inactivated polio vaccine.

PCV 13: Pneumococcal 13-valent conjugate vaccine.

ROTA: Rotavirus vaccine.

MCV: Measles-containing vaccine.

YF: Yellow fever vaccine.

## 4 Discussion

This study assessed the usability and the quality of HBR photos and found that the majority of HBR photos included the basic elements of an immunization record and were completed in French, the national language. Most HBR photos indicated that official Ministry of Health cards were the most used variation, with the national immunization schedule included at the top, as well as separate spaces for each immunization date. Nearly all cards contained information on vaccines recommended as a part of the national immunization schedule. However, the variations in the proportion vaccinated seen from card transcription in the field and at the central level from HBR photos is very concerning. This study was not designed to explore why these differences in central or field-recorded data exist, and thus this needs further exploration.

HBR photos provide basic elements of a vaccination card including among other basic demographics such as the child's name, date, and place of birth, and sex [8]. The presence of these elements in HBR photos is considered necessary for assessing immunization records and functions related to immunization services. These results are similar to those reported by Harrison et al. [17] in South Africa; yet, the birth weight of the child was indicated on the HBR in DRC less frequently than in the South African cohort, 76.0 % and 94.0 %, respectively. Of note, only 32.1 % of cards were completed with the national registration number for the child – this information is essential for social services. Without an official birth registration, the child cannot be issued an official birth certificate, and this is considered a legal obligation in the DRC under the Code of the Congolese Family [18,19].

The good quality of the HBR photos allowed easy transcription of all the information contained in the card. Image obstruction by a foreign object led to a loss of information included on the card, and the presence of a sign of physical damage was likely to reduce the possibility of reading the information on the card. In this study, in the majority of HBR photos analyzed, the image quality was good, with a foreign object obstructing about 30 % of the time, but only 3 % impacting the information needed. For most cards, there were no signs of physical damage (80 %). This is aligned with a similar study conducted in Lebanon, which showed that the image quality was high, with only a few images obstructed by a foreign object and less than 3 % of images considered too blurry, overexposed, or distorted to be readable. Moreover, more than 85 % of the cards had no visible physical damage, nor any obstruction in the form of marks, perforations, or staples that could have hindered the readability of the information on the vaccination card [14].

More than 90 % of the cards had enough space available to note the date of the next routine immunization visit. This aligns with the results from Lebanon, which showed that all cards (*n* = 500) included information on vaccines recommended according to the national immunization schedule [14]. The cards had sufficient space available for the date of receiving each vaccine and dose (78.6 %). Only some cards included space for the next routine immunization visit and space for healthcare provider narrative notes. In this study, the majority of cards prominently displayed the date of the next routine immunization visit (83.9 %). The study showed that HBR photos were legible as handwriting contained in the cards were read without problems (>70.0). This percentage is slightly higher than that found in the study conducted in Lebanon, which showed that the handwriting on the forms was deemed acceptable for only slightly over half of the card photos [14].

Each HBR photo contained information on vaccines recommended by the national immunization schedule that were administered to the child according to healthcare providers and their data of administration. The legibility of this data allowed their recording and comparison with vaccine data entered during household surveys. The agreement assessment between data from HBR photos and 2022 VCS data suggested that data collectors in the fieldwork were able to accurately recorded child's immunization status, yet this data accuracy may be worse for vaccines that require multiple doses. The kappa agreement suggests that one of the groups involved in data collection did not properly record data. Although the group that entered at the central level worked with fewer constraints, it is possible that the quality of the photos could work against them and reduce available data if photo quality made the data impossible to enter. Conversely, when entering the HBR at the time of the survey, the interviewer may be able to verify immunization status with the family and can verify with the physical document as opposed to those using an HBR photo which may not be well captured or have inconsistent information. A more complete comparison on the correctness of card entry in a central location compared to during the survey data collection time should be completed.

This study has limitations. The proportion of cards seen in the VCS is low and only a small subset (6.7 %) were included in this analysis for central entry. Data were collected by observation done by only one data collector and the study does not use double observation to check the concordance. Also, the aspect of time that taken to extract data in the field versus from pictures was not assessed, which would have been useful to evaluate benefits of each approach. It would be beneficial if the data were verified by a second observer in order to increase confidence in the data observation. This work will be explored separately.

## 5 Conclusion and recommendations

This study demonstrates that using HBR photos at a central level taken during a coverage survey is a feasible and potentially useful approach to conduct vaccine coverage analyses and HBR evaluations, such as assessing their quality. Results from this study suggest the need to maintain healthcare workers' awareness about the importance of understandable and standardized record keeping, as well as reminding parents to keep HBRs. The standardized usage will facilitate the utilization of cards by different healthcare workers and will prevent potential misunderstandings on card content and more card availability will result in more accurate coverage evaluation survey results. Therefore, future studies are needed to examine the HBR content, specifically the way health providers fill out the HBR and the accuracy of vaccine data.

## CRediT authorship contribution statement

**Dorcas M. Kibeti:** Writing – review & editing, Writing – original draft, Investigation, Formal analysis, Conceptualization. **Nicole A. Hoff:** Writing – review & editing, Writing – original draft, Resources, Project administration, Methodology, Investigation, Conceptualization. **Sydney Merritt:** Writing – review & editing, Writing – original draft, Project administration, Methodology, Formal analysis, Conceptualization. **Aimée M. Lulebo:** Writing – review & editing, Resources, Project administration, Methodology, Investigation, Data curation, Conceptualization. **Jean-Bosco N. Kasonga:** Writing – review & editing, Project administration, Methodology, Investigation, Data curation. **Nono M. Mvuama:** Writing – review & editing, Supervision, Resources, Project administration, Methodology, Investigation, Conceptualization. **Christophe L. Luhata:** Writing – review & editing, Supervision, Resources, Investigation, Conceptualization. **Dalau M. Nkamba:** Writing – review & editing, Supervision, Project administration, Methodology, Investigation, Formal analysis, Data curation, Conceptualization. **John Samuel Otomba:** Writing – review & editing, Supervision, Methodology, Investigation, Funding acquisition, Conceptualization. **Amine El Mourid:** Writing – review & editing, Supervision, Resources, Methodology, Investigation, Funding acquisition, Conceptualization. **Aimé M.W. Cikomola:** Writing – review & editing, Project administration, Methodology, Conceptualization. **Jean-Crispin Mukendi:** Writing – review & editing, Supervision, Resources, Investigation, Conceptualization. **Marcellin Mengouo Nimpa:** Writing – review & editing, Supervision, Methodology, Funding acquisition, Conceptualization. **Daniel K. Ishoso:** Writing – review & editing, Project administration, Methodology, Conceptualization. **Adèle N. Mudipanu:** Writing – review & editing, Supervision, Methodology, Conceptualization. **Deo Manirakiza:** Writing – review & editing, Supervision, Methodology, Conceptualization. **Anne W. Rimoin:** Writing – review & editing, Supervision, Resources, Project administration, Funding acquisition, Conceptualization. **Didine K. Kaba:** Writing – review & editing, Supervision, Resources, Project administration, Methodology, Funding acquisition, Conceptualization. **Jean K. Nyandwe:** Writing – review & editing, Supervision, Resources, Methodology, Conceptualization. **M. Carolina Danovaro-Holliday:** Writing – review & editing, Supervision, Methodology, Investigation, Conceptualization. **Paul-Samson D. Lusamba:** Writing – review & editing, Supervision, Resources, Project administration, Methodology, Funding acquisition, Conceptualization. **Eric M. Mafuta:** Writing – review & editing, Writing – original draft, Supervision, Resources, Methodology, Investigation, Funding acquisition, Formal analysis, Data curation, Conceptualization.

## Disclaimer

MCD-H works at the World Health Organization. The comments on this article reflect those of the author alone and do not necessarily reflect those of the World Health Organization.

## Funding

This 2022 DRC VCS survey from which this study is drawn was funded by the Bill & Melinda Gates Foundation, GAVI Alliance, UNICEF and USAID through the United Nations Children's Fund. Additional support from the 10.13039/100000865Bill and Melinda Gates foundation was provided under grant number INV-035951.

## Declaration of competing interest

The authors declare the following financial interests/personal relationships which may be considered as potential competing interests: Anne W. Rimoin reports financial support was provided by Bill and Melinda Gates Foundation. If there are other authors, they declare that they have no known competing financial interests or personal relationships that could have appeared to influence the work reported in this paper.

## Data Availability

Data will be made available on request.
